# Stimulation of follicle growth and development during estrus in Ettawa Grade does fed a flushing supplement of different polyunsaturated fatty acids

**DOI:** 10.14202/vetworld.2021.11-22

**Published:** 2021-01-05

**Authors:** Prasetyo Nugroho, Komang Gede Wiryawan, Dewi Apri Astuti, Wasmen Manalu

**Affiliations:** 1 Graduate School of Nutrition and Feed Science, Faculty of Animal Science, IPB University, Jalan Agatis, Kampus IPB Dramaga, Bogor 16680, Indonesia; 2 Department of Livestock and Animal Health Services of Central Java Province, Jalan Jenderal Gatot Subroto, Tarubudaya, Ungaran 50517, Indonesia; 3 Department of Nutrition and Feed Technology, Faculty of Animal Science, IPB University, Jalan Agatis, Kampus IPB Dramaga, Bogor 16680, Indonesia; 4 Department of Anatomy, Physiology, and Pharmacology, Faculty of Veterinary Medicine, IPB University, Jalan Agatis, Kampus IPB Dramaga, Bogor 16680, Indonesia

**Keywords:** Ettawa Grade does, flushing, follicle, lauric acid, linoleic acid, α-linolenic acid

## Abstract

**Background and Aim:**

Flushing with the manipulation of fatty acids, particularly polyunsaturated fatty acids, like linoleic and α-linolenic acids in the ration, is a strategy to raise the nutritional status of the female mammals to improve ovarian function and follicle development. This study was designed to investigate the effectiveness of flushing supplementation with different types of polyunsaturated and saturated fatty acids in stimulating follicle growth and development during estrus in Ettawa Grade does with a low initial body condition score (BCS ≤2).

**Materials and Methods:**

Eighteen Ettawa Grade does in the second parity, with an average body weight of 32.11±2.19 kg, were divided into three groups according to the fatty acid supplemented to their ration: (i) About 2.8% lauric acid flushing (group); (ii) 2.8% linoleic acid flushing (LA group); and (iii) 2.8% α-linolenic acid flushing (ALA group). The ration was formulated to be isocaloric (total digestible nutrient = 77%) and isonitrogenous (crude protein = 15%). The experiment was conducted for 35 days; that is, 14 days for acclimatization and synchronization of the estrous cycle and 21 days for fatty acid flushing until the appearance of the next estrus. A completely randomized design was applied.

**Results:**

According to the results, none of the different fatty acids in the ration affected the nutrient intakes, BCSs, average daily gains, and plasma glucose, cholesterol, and progesterone concentrations of the three groups of does. However, the BCSs (by 0.8-0.9) and the plasma cholesterol concentrations were higher after fatty acid flushing for 21 days than before the flushing period. The ALA group had the highest number of large-sized preovulatory follicles, whereas the LAURIC group had the highest plasma estradiol concentration during estrus. All three groups had similar plasma progesterone concentrations during estrus after fatty acid flushing.

**Conclusion:**

Flushing supplementation with 2.8% ALA from flaxseed oil gave the best results in terms of stimulating the highest number of large-sized preovulatory follicles in Ettawa Grade does.

## Introduction

The Ettawa Grade goat is a dual-purpose breed (i.e., intended for meat and milk production) that has adapted well to humid tropical conditions. In Indonesia, although these goats are prolific, they are less prolific than Kacang goats, the other local goat breed [1]. Moreover, goat breeding in Indonesia is still mostly carried out on a small scale, with the majority of operations being smallholder farms. The ration provided by such farms tend to be poor in nutrition, resulting in animals with a low body condition score (BCS ≤2), which can reduce their reproductive performance. Therefore, there is a need to improve and to enhance the reproductive functions of Ettawa Grade goats in Indonesia. It is known that the reproductive performance of ruminants can generally be improved through a nutritional approach of flushing supplementation [2]. As concluded in the previous studies, nutrition plays an important role in influencing the reproductive capacity and ovarian activity of does, where improvement of the ration quality can increase the ovulation rate [3,4]. So far, the supplementation of carbohydrates and fats into the ration formulation has been used to improve the ovarian function and reproductive capacity of small ruminants [5]. There is consensus among the scientific communities that the effect of fat supplementation on reproduction is due to the increased energy status of the livestock and the essential roles that fatty acids play in determining the reproductive performance of the animal [6]. Since fatty acids are structural and functional ­components of cell membranes, dietary supplementation with polyunsaturated fatty acids (PUFAs) can affect the cell membrane composition [7] as well as various biological processes, such as membrane fluidity, nutrient transport, and membrane-attached enzyme activity [8].

Several studies have considered the utilization of different PUFA sources for improving reproductive functions in ruminants [2,9-12]. Alpha (α)-linolenic acid supplementation at a dose of 100 mM during *in vitro* maturation was reported to increase the nuclear maturity rate of buffalo oocytes [13]. Compared with those fed a diet supplemented with saturated fatty acids (SFAs), dairy cows fed a diet supplemented with flaxseed oil showed increased folliculogenesis [14]. Furthermore, the supplementation of ewes with omega-3 PUFAs from fish oil improved the growth of their follicles and corpus luteum and increased the synthesis and secretion of estrogen and progesterone [12], which ultimately improved the quality, growth, and development of the embryos [15,16]. The dietary supplementation of α-linolenic acid (ALA) increases its conversion into eicosapentaenoic acid (EPA), which leads to the formation of 3-series eicosanoids and increases the synthesis and secretion of progesterone [6]. However, the previous studies have indicated that ALA or omega-3 is not consistently better than linoleic acid (LA) or omega-6 in improving the number of large-sized preovulatory follicles [9,10]. Petit *et al*. [9] reported that there was no significant difference in the number of large-sized follicles between dairy cows fed an LA-supplemented diet and those fed an ALA-supplemented one. This finding was corroborated by another study, which showed that the number and size of follicles were not different between ewes fed a diet supplemented with LA and those fed a diet supplemented with ALA, but the concentration of progesterone was higher in the latter group [10]. The inconsistent effects of the sources of omega-3 supplementation on the follicular response may have been due to the non-comparable levels of omega-3 and omega-6 supplemented or the difference in palatability of the fat sources (i.e., animal fats vs. plant oils). Therefore, in comparison studies, the fatty acid content for fat supplementation should be adjusted. Furthermore, a previous study conducted in our laboratory by Khotijah *et al*. [11] showed that improvement of the ration quality through supplementation with 4% sunflower oil as the LA source increased the reproductive performance of ewes relative to that obtained with no oil supplementation. However, to the best of our knowledge, there have been no *in vivo* studies conducted on the manipulation of fatty acids in the diet during the flushing period or comparisons of the effectiveness of omega-3 and omega-6 supplementation at the same level in stimulating follicle development in goats in Southeast Asia, a tropical region with a humid environment.

Therefore, this study was carried out to compare the effectiveness of PUFA (LA or ALA) or SFA (lauric acid [LAURIC]) flushing supplementation in stimulating follicle growth and development during estrus in Ettawa Grade does with a low initial BCS (≤2) in Southeast Asia.

## Materials and Methods

### Ethical approval

Ethical clearance was provided by the Animal Care and Use Committee of IPB University (approval number: 119-2018).

### Study period and location

The animal experiments were performed from March to April 2018 at the Field Laboratory of Meat and Draught Animal Nutrition, IPB University, Bogor, Indonesia.

#### Experimental animals, diets, and protocol

Eighteen Ettawa Grade does in the second parity, with an average body weight (±SD) of 32.11 (±2.19) kg and an initial BCS of 1.85 (±0.30), belonging to the Field Laboratory of Meat and Draught Animal Nutrition, IPB University, Bogor, Indonesia, were used.

The does were randomly divided into three groups: (i) Those fed ration supplemented with 2.8% LAURIC from coconut oil (Barco^®^, PT. Barco, Jakarta, Indonesia) (LAURIC group; n=6); (ii) those fed ration supplemented with 2.8% LA from sunflower oil (Mazola^®^, Moi Foods Malaysia Sdn Bhd, Selangor, Malaysia) (LA group; n=6); and (iii) those fed ration supplemented with 2.8% ALA from flaxseed oil (Green Tosca^®^, Proteco Gold Pty Ltd, Kingaroy, Australia) (ALA group; n=6). The fatty acid profiles of the coconut oil, sunflower oil, and flaxseed oil are presented in Table-1. The isocaloric (total digestible nutrient [TDN]=77%) and isonitrogenous (crude protein [CP]=15%) ration contained Napier grass forage and concentrate in the dry matter ratio of 30:70. The composition and nutrient contents of the ration are presented in Table-2. The concentration of mixed diet provided to the does was 3.5% of total body weight, whereas drinking water was consumed ad libitum.

**Table-1:** Composition of the fatty acid (%) contents in coconut oil, sunflower oil, and flaxseed oil.

Fatty acid	Coconut oil	Sunflower oil	Flaxseed oil
Caprylic acid (C8:0)	6.70	—	—
Capric acid (C10:0)	5.26	—	—
Undecanoic acid (C11:0)	0.02	—	—
Lauric acid (C12:0)	45.74	0.02	0.03
Myristic acid (C14:0)	16.24	0.06	0.06
Palmitic acid (C16:0)	7.23	5.13	4.93
Palmitoleic acid (C16:1)	—	0.08	0.24
Heptadecanoic acid (C17:0)	—	0.03	0.04
*cis*-10-Heptadecanoic acid (C17:1)	—	—	0.03
Stearic acid (C18:0)	1.96	2.44	4.00
Oleic acid (C18:1 n-9)	4.87	27.59	18.33
Linoleic acid (C18:2 n-6)	1.27	59.83	15.83
Arachidic acid (C20:0)	0.04	0.17	0.13
α-Linolenic acid (C18:3 n-3)	0.04	0.47	53.97
*cis*-11,14-Eicosadienoic acid (C20:2)	—	0.06	0.06
Behenic acid (C22:0)	—	0.45	0.03
Tricosanoic acid (C23:0)	—	0.02	0.04
Lignoceric acid (C24:0)	0.02	0.15	0.06

**Table-2:** Ingredients and nutrient contents of the fatty acid-supplemented ration (dry matter basis) fed to Ettawa Grade does.

Variable	Group		

	LAURIC	LA	ALA
Ingredients (% DM)			
Napier grass	30.00	30.00	30.00
Soybean meal	14.25	14.15	14.15
Corn gluten feed	19.31	19.31	19.31
Dried cassava	29.40	30.90	30.40
CaCO	0.32	0.32	0.32
Premix*	0.32	0.32	0.32
Salt	0.30	0.30	0.30
Coconut oil	6.10	—	—
Sunflower oil	—	4.70	—
Flaxseed oil	—	—	5.20
Nutrient contents (% DM)			
Crude protein	15.26	15.25	15.24
Ether extract	7.51	6.12	6.62
Crude fiber	11.18	11.20	11.19
TDN	77.19	76.74	76.91
Calcium	0.57	0.57	0.57
Phosphorus	0.37	0.37	0.37

DM=Dry matter, TDN=Total digestible nutrient, LAURIC=6.1% coconut oil containing 2.8% lauric acid, LA=4.7% sunflower oil containing 2.8% linoleic acid, ALA=6.1% flaxseed oil containing 2.8% α-linolenic acid.

* Each 1 kg premix contained vitamins A (2,000,000 IU), D (400,000 IU), E (600 mg), B1 (200 mg), B2 (1000 mg), B12 (1,000 mcg), and K (200 mg); niacinamide (1500 mg); calcium d-pantothenate (500 mg); folic acid (100 mg); choline chloride (20,000 mg); dl-methionine (20,000 mg); l-lysine (15,000 mg); magnesium sulfate (6,800 mg); ferrous sulfate (5000 mg); manganese sulfate (10,000 mg); cupric sulfate (1000 mg); zinc sulfate (2000 mg); and potassium iodide (20 mg)

**Figure-1:**
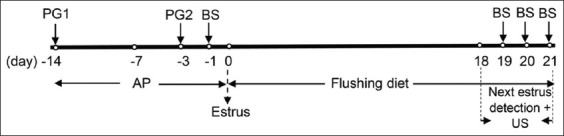
Protocol of the experiment from acclimatization, flushing, estrus synchronization, detection of the next estrus cycle, blood sample collection, and ultrasonography. AP=Acclimatization period; BS=Blood sampling; PG=Prostaglandin F_2_^α^ injection; US=Ultrasonographic scanning.

The experiment was conducted for 35 days, 14 days of which were for acclimatization and estrus synchronization (Figure-1). Flushing supplementation was carried out for 21 days, starting from 3 days after estrus synchronization until the appearance of the next phase of the estrous cycle after the flushing period. The feed intake during fatty acid flushing was measured by subtracting the dry matter feed residue from the dry matter feed offered.

The BCS of each doe was measured 1 day before flushing (day −1), to obtain the pre-flushing baseline value, and then again on day 21 after flushing. The BCS was assigned a value ranging from 1 to 5 through assessment of the fat on the animal’s backbone and hip by pressing with the fingertips and hands, as described by Koyuncu and Altınçekiç [17]. A BCS of one indicates an extremely thin doe; that is, the backbone and ribs are clearly visible, the flank is hollow, there is no fat cover, and the examiner’s fingers can penetrate the intercostal spaces without difficulty. A BCS of two indicates a thin doe; that is, the backbone is still visible as a continuous ridge, several ribs can be seen and felt, there is a small amount of fat cover, and the intercostal spaces are smooth but still penetrable. A BCS of three indicates a doe of normal weight; that is, the backbone is not prominent, the ribs are barely discernible and are covered by an even layer of fat, and the intercostal spaces can only be felt using pressure. A BCS of four indicates a fat doe; that is, the backbone and ribs are invisible, and the outer body of the animal is sleek in appearance. A BCS of five indicates an obese doe; that is, the backbone of the doe is buried in fat, and the ribs are invisible and covered with excess fat.

### Estrus synchronization and ultrasonographic examination

To ensure that the does were in the same phase of estrus before the flushing experiment, their estrous cycles were first synchronized by injecting each of them intramuscularly with prostaglandin F2 alpha (PGF2α) in the form of 5 mg dinoprost tromethamine (Lutalyse^®^, Zoetis Ireland Ltd., Dublin, Ireland), twice at an interval of 11 days. The first PGF2α injection was administered on day 0 of acclimatization (14 days before flushing), and the second injection was administered on day 11 (3 days before flushing). Three days after the second PGF2α injection, the does were detected to be in estrus on the same day, which was designated as the day for initiating flushing (day 0). The estrous phase of the does after flushing was detected from their interactions with a buck.

The development of the follicles and corpus luteum after flushing was monitored by ultrasonography (SSD-500, ALOKA, Tokyo, Japan) using a linear probe (frequency of 7.5 MHz). The ovaries at the estrous phase were also scanned by ultrasonography on each morning of days 18-21 of the flushing period (Figure-1) for the enumeration of each distinct class of follicles. The follicles were measured and grouped into three classes according to their diameter size: Small (2-3 mm), medium (3.1-5 mm), and large (>5 mm). In addition, the preovulatory follicle number was determined on the day before ovulation (day 20 or 21 of flushing) (Figure-1). Ovulation was determined by the development of a corpus luteum in the ovary. The follicle numbers and diameters were acquired and evaluated daily during the estrous phase for the observation of follicular development and were also evaluated at preovulation.

### Blood sampling

On days −1, 19, 20, and 21 of flushing, blood samples were collected through the jugular vein using a syringe (5 mL) and transferred into EDTA glass tubes. Plasma was collected after centrifugation of the blood sample at 3000 rpm for 15 min and stored at −20°C until assayed. The day −1 plasma sample was used for cholesterol and glucose measurements; the day 19 plasma sample was used for cholesterol, glucose, and estradiol measurements; and the day 20 and day 21 plasma samples were used for progesterone measurement.

### Analysis of plasma concentrations of cholesterol, glucose, estradiol, and progesterone

The plasma cholesterol concentration was analyzed using a cholesterol assay kit (Cat No. 101592 Reg. No. AKL 10101803466; PT. Rajawali Nusindo, Jakarta, Indonesia). The plasma glucose concentration was analyzed using a glucose assay kit (Cat Nos. 112191 and 112192 Reg. No. AKL 20101803460; PT. Rajawali Nusindo). The plasma estradiol and ­progesterone concentrations were quantified by enzyme-linked immunosorbent assay, according to the instructions accompanying the Estradiol-DRG EIA 2693 Kit (DRG Instruments GmbH, Marburg, Germany) and Progesterone-DRG EIA 1561 Kit (DRG Instruments GmbH), respectively.

### Statistical analysis

This study used a completely randomized design. The BCS, plasma glucose concentration, and plasma cholesterol concentration data generated from repeated samples were analyzed by repeated-measures analysis of variance (ANOVA) [18]. Data on the nutrient intake, final live weight, average daily gain (ADG), follicle number and diameter, corpus luteum diameter, plasma estradiol concentration, and plasma progesterone concentration were analyzed using the completely randomized design ANOVA, followed by Duncan’s multiple-range test [19]. Correlations between the total follicle number and plasma estradiol concentration and between the corpus luteum diameter and plasma progesterone concentration were analyzed using correlation analysis. All analyses were carried out using SAS software (version 9.4) under IPB University license.

## Results

### Feed intake, ADG, and BCS

The nutrient intakes of does fed diets supplemented with different types of fatty acids are presented in Table-3. The different types of fatty acids did not significantly (p>0.05) affect the animal intakes of dry matter, TDN, CP, fats, and fiber. For all three groups of does, the range of dry matter intake (DMI) during the flushing period was 873.84-886.17 g/head/day or 2.66-2.71% of body weight (Table-3). Moreover, flushing did not influence the calcium and phosphorus intakes, regardless of the type of fatty acid supplemented.

**Table-3:** Nutrient intake and performance of does fed diets supplemented with lauric acid, linoleic acid, or α-linolenic acid during the flushing period.

Variable	Group		

	LAURIC	LA	ALA
Nutrient intake			
Dry matter (g/head/day)	873.84±90.59	881.43±154.80	886.17±58.12
Crude protein (g/head/day)	133.35±13.82	134.42±23.61	135.05±8.86
Ether extract (g/head/day)	65.63±6.80	53.94±9.47	58.66±3.85
Crude fiber (g/head/day)	97.70±10.13	98.72±17.34	99.16±6.50
TDN (g/head/day)	674.52±69.93	676.41±118.79	681.55±44.70
Calcium (g/head/day)	4.98±0.52	5.02±0.88	5.05±0.33
Phosphorus (g/head/day)	3.23±0.34	3.26±0.57	3.28±0.22
Dry matter (% body weight)	2.66±0.19	2.66±0.31	2.71±0.19
Performance			
Initial live weight (kg)	32.18±2.69	32.15±2.38	32.02±1.86
Final live weight (kg)	34.23±2.76	34.07±2.55	34.03±1.81
Live weight gain (kg)	2.05±0.15	1.98±0.12	2.02±0.15
Average daily gain (g/day)	97.62±11.57	94.44±11.03	96.03±7.01

TDN=Total digestible nutrient, LAURIC=Lauric acid, LA=Linoleic acid, ALA=α-linolenic acid

The live weight reached after fatty acid flushing was similar in all three groups (p>0.05), ranging from 34.03 to 34.23 kg (Table-3). The increase in live weight during the 21 days of flushing was 1.98-2.05 kg for all groups. This was equivalent to an ADG of 91.27-97.62 g, which was also not influenced by the fatty acids in the flushing diet (p>0.05).

**Figure-2:**
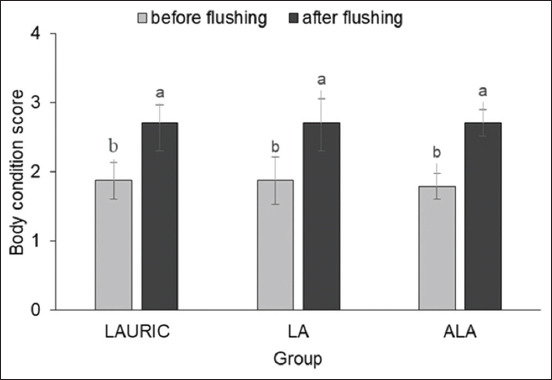
Body condition score of does before and after flushing with ration supplemented with lauric acid or linoleic acid, and or α-linolenic acid. ^ab^Different letters above the data in the same group indicate significant difference (p<0.01) means for before and after flushing.

The BCSs of the does before flushing were low and similar among all three groups (~1.8; p>0.05). The mean BCSs were still similar across all groups after 21 days of flushing (2.71±0.32; p>0.05). However, the BCSs before flushing were significantly lower than those after the 21 days of flushing (p<0.01), with the increase ranging from 0.8 to 0.9 (i.e., 1.8 before flushing to 2.71 after flushing) (Figure-2).

### Numbers and diameters of the follicles and corpus luteum

**Figure-3:**
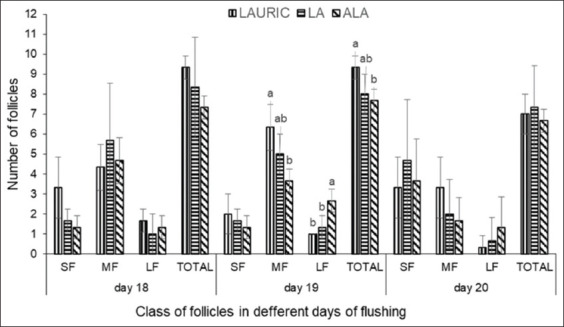
The number of small follicles, medium follicles, large follicles, and total follicles in does fed the flushing diet supplemented with lauric acid or linoleic acid, and or α-linolenic acid. SF=Small follicles (2-3 mm); MF=Medium follicles (3.1-5 mm); LF=Large follicles (LF >5 mm). ^ab^Different letters above the data in the same size of follicle indicate a significant difference (p<0.05).

Figure-3 presents the mean numbers of follicles during estrus in the three groups after fatty acid flushing. On days 18 and 20 of flushing supplementation, no significant effects (p>0.05) on the number of small, medium, and large follicles and on the total number of follicles were observed. However, on day 19 of flushing, the ALA group had the highest number (p<0.05) of large follicles. By contrast, on the same day, the LAURIC group had a higher number (p<0.05) of medium and total follicles than had the ALA group. However, there was no significant difference (p>0.05) in the numbers of medium and total follicles between the LAURIC and LA groups and between the LA and ALA groups.

**Figure-4:**
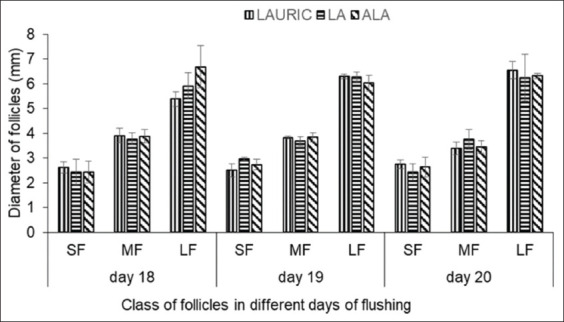
The diameter of small follicles, medium follicles, and large follicles in does fed flushing diet supplemented with lauric acid or linoleic acid, and or α-linolenic acid. SF=Small follicles (2-3 mm); MF=Medium follicles (3.1-5 mm); LF=Large follicles (LF >5 mm).

Figure-4 presents the mean follicle diameters during estrus in the three groups after flushing supplementation. As determined on days 18, 19, and 20 of the flushing period, the diameters of the small, medium, and large follicles were not significantly different (p>0.05) among all the groups.

On the day before ovulation (day 20 or 21 of flushing), the numbers of large preovulatory follicles in the animals of the LAURIC, LA, and ALA groups were 1.00±0.00, 1.33±0.57, and 2.67±0.58, respectively, with the high number in the ALA group being statistically significant (p<0.05). However, the difference in the numbers of large preovulatory follicles between the LAURIC and LA groups was not statistically significant (p>0.05). The post-ovulatory corpus luteum was clearly still a corpus hemorrhagicum, with diameters of 6.60±0.53, 6.45±0.44, and 6.80±0.45 mm in the LAURIC, LA, and ALA groups, respectively, and not statistically significantly different (p>0.05) among the groups. Although the diameters of all classes of follicles and the corpus luteum were not significant, that of the corpus luteum tended to be larger in the ALA group (p=0.68) than in the LAURIC and LA groups.

### Plasma metabolites and steroid hormones

**Figure-5:**
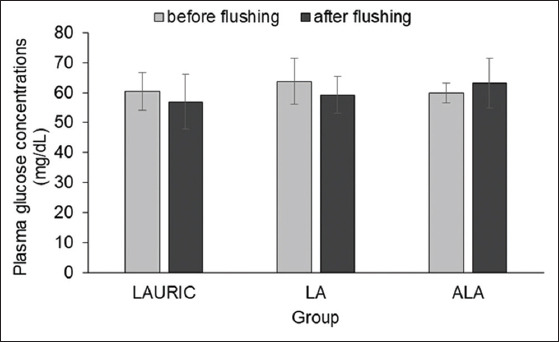
Plasma glucose concentrations in Ettawa Grade does fed ration supplemented with lauric acid or linoleic acid, and or α-linolenic acid before and after the flushing period.

**Figure-6:**
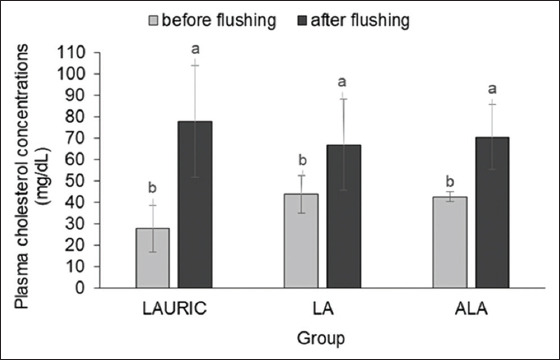
Plasma cholesterol concentrations in Ettawa Grade does fed ration supplemented with lauric acid or linoleic acid, and or α-linolenic acid before and after the flushing period. ^ab^Different letters above the data in the same group of flushing supplements indicate a significant difference (p<0.05).

Among the animals in all three groups, the plasma glucose concentrations were similar before and after the flushing period (59.81±7.42 vs. 61.34±5.55 mg/dL; p>0.05) (Figure-5). By contrast, although the mean plasma cholesterol concentrations were not significantly different (p>0.05) among all three groups after the flushing period (Figure-6), the values were significantly higher (p<0.05) than those before flushing (Figure-6), regardless of the fatty acid supplemented.

The mean plasma estradiol concentration in does of the LAURIC group was significantly higher (p<0.05) than that in does of the LA and ALA groups (Table-4). However, the values of the LA and ALA groups were similar (p>0.05).

**Table-4:** Plasma estradiol and progesterone concentrations in Ettawa Grade does fed diets supplemented with lauric acid, linoleic acid, or α-linolenic acid during the estrous cycle.

Variable	Group		

	LAURIC	LA	ALA
Plasma estradiol concentration (pg/mL)	176.37±56.77^a^	69.10±1.45^b^	67.63±9.72^b^
Plasma progesterone concentration (ng/mL)	0.29±0.14	0.19±0.05	0.40±0.24

^ab^Different superscripted letters in the same row indicate a significant difference (p<0.05). LAURIC=Lauric acid, LA=Linoleic acid, ALA=α-linolenic acid

The plasma progesterone concentrations during estrus were similar among all three groups after flushing (p>0.05) (Table-4). However, the concentrations tended to be higher in the ALA group than in the other two groups (p>0.05) (Table-4).

### Correlations between the total number of follicles, diameter of the corpus luteum, and steroid hormones

Table-5 presents the correlations between the total follicle numbers and plasma estradiol concentrations and between the corpus luteum diameters and plasma progesterone concentrations. There was a strong positive correlation between the total number of follicles and the concentration of plasma estradiol in the LAURIC and ALA groups but only a moderate positive correlation in the LA group. A strong positive correlation existed between the diameter of the corpus luteum and the concentration of plasma progesterone in the LAURIC group, whereas the positive correlation was moderate in the LA and ALA groups. The correlations observed in each group were not significant (p>0.05) due to the low number (n=6) of replications in each group. However, when the correlations were assessed on the basis of the overall 18 replications among all three groups, they were significant (p<0.05). That is, a strong positive association between the total number of follicles and the concentration of plasma estradiol and a moderate positive association between the diameter of the corpus luteum and the concentration of plasma progesterone were found in the does overall.

**Table-5:** Correlations between the total number of follicles and the concentration of plasma estradiol and between the diameter of the corpus luteum and the concentration of plasma progesterone in Ettawa Grade does fed diets supplemented with LAURIC, LA, or ALA during the estrous cycle.

Variable	Group			

	LAURIC (n=6)	LA (n=6)	ALA (n=6)	Overall (n=18)
Total number of follicles and concentration of plasma estradiol	0.776 (p=0.313)	0.534 (p=0.478)	0.754 (p=0.330)	0.631* (p=0.011)
Diameter of corpus luteum and concentration of plasma progesterone	0.819 (p=0.280)	0.598 (p=0.437)	0.571 (p=0.454)	0.550* (p=0.022)

* Significant difference (p<0.05). LAURIC=Lauric acid, LA=Linoleic acid, ALA=α-linolenic acid

## Discussion

Flushing is a new agricultural technique that focuses on increasing the energy density of the feed provided to animals before mating. In this study, it was applied to determine which type of fatty acids could stimulate follicle growth and development during estrus in Ettawa Grade does. We found that supplementation of fats (in the range of 4.7-6.1%) with different fatty acid profiles to the ration had no adverse effect on the DMI during the flushing period, implying that the palatability of the experimental diets was excellent. Similar results were observed by other researchers, who found that the supplementation of 5% fat with different ratios of omega-3 and omega-6 to the diets of ewes did not affect the DMI [20]. In another study, although the supplementation of up to 6% sunflower oil in the diet did not affect the DMI, it did negatively influence the rumen fermentability of lactating Garut ewes [21]. Since the diets in our study were formulated to be isocaloric and isonitrogenous, the similar DMI% among the groups confirmed that the does did indeed obtain the same levels of energy and protein to be used for reproduction. The TDN and CP intakes in this study were higher than those recommended by the US National Research Council (470 and 59 g/head/day, respectively) [22] for the breeding of does with a body weight of 30 kg. This was likely due to the flushing supplementation of diets containing high energy and protein, where the excess energy intake mostly accumulated in adipose tissue, providing increased fat deposition to improve the BCS for reproduction.

Similar to the DMI, there was no significant impact on the live weight gain and BCS by any of the fatty acid supplementations. In this study, a BCS increase of 1-point was equivalent to 2.24-2.56 kg of live weight gain. Similarly, Aidismen *et al*. [23] reported that a 1-point change in the BCS of Ettawa Grade does produced live weight gains of 2.61-3.25 kg. However, the value in our study was lower than reported by Sanson *et al*. [24], where a 1-point change in BCS equated to a 5.1-kg change in the live weight of ewes. The possible explanations for the variations in live weight gain per 1 BCS point are the difference in breeds used among the various studies and the differences in their body sizes and conformation and fat distribution throughout the animal [25].

The BCS can be used as an indicator of the nutritional status of the animal. Given that it has been shown that a BCS in the range of 2.5-3.0 at the time of mating would optimize the profitability of Merghoz goats and their kids [26], it stands to reason that the improved BCS of does with an initial BCS of <2 from 21 days of fatty acid flushing would significantly support their reproductive process and increase their profitability as well. Several studies have reported similar improvements to the BCS of small ruminants through flushing supplementation with different fatty acids [3,12,27]. For example, the increased intake of nutrients (especially energy) by Spanish×Boer crossbred does improved their body conditions, as reflected by the increased BCS values, allowing the animals to begin the reproductive process (including pregnancy, giving birth, and suckling) [28]. Flushing also decreased shrinkage of the ewe body weight at postpartum [21]. The improved BCS by the fatty acid flushing provides signals of the readiness of the body system to enter the reproductive process. The flushing treatment will increase the number and size of adipocytes, which signals them to increase leptin synthesis. This is supported by the findings of a positive correlation between the number of adipocytes and the concentration of leptin [29] and that leptin is synthesized by adipose cells [30]. The accumulated leptin molecules eventually act directly on the hypothalamus to raise the pulsatile secretion of gonadotropin-releasing hormone, which stimulates and increases the synthesis and secretion of luteinizing hormone [31]. The increased secretion of luteinizing hormone stimulates the production of progesterone by the corpus luteum, leading to an increase in the circulating progesterone level [32]. In this study, the nutrient intake and BCS trends were similar, clearly indicating that follicle development and steroidogenesis were affected by specific fatty acids.

The similar plasma glucose concentrations before and during the flushing period were caused by the increased utilization of glucose for thermoregulation. Fat supplementation generally does not alter glucose concentrations in the blood [33]. In addition to its role as a primary metabolic fuel for the central nervous system, glucose serves as a primary energy source for the ovary during the reproductive stage [34].

The finding of similar plasma cholesterol concentrations in all three groups is similar to that reported by Kia and Safdar [2]. The increase in plasma cholesterol concentrations in the fatty acid-supplemented does is associated with the increased intake of crude fat in the flushing period. Although the concentrations of plasma cholesterol were similar in all three groups during flushing, the animals supplemented with the SFA (LAURIC) had the highest levels. The high amount of crude fat taken in during the flushing period is used not only as an energy source but also as a precursor of cholesterol. The increased plasma cholesterol molecules in the does are used to build the cell membrane and as precursors for estradiol synthesis [35].

The findings of this study suggest that follicle growth and development are affected not only by fat supplementation but also by the type of fatty acids in those fats. Different fatty acid types had different effects on the development of follicles and steroidogenesis [7]. Flushing with 2.8% ALA had a significant effect in stimulating preovulatory follicle development to a larger size before ovulation, where the number of large preovulatory follicles was the highest in the ALA group, with statistical significance. However, the plasma estradiol concentration during estrus was lower in this group than in the LAURIC group. In fact, a significant increase in the estradiol concentration during estrus occurred only in the LAURIC group.

Fatty acid supplementation to the diet of ruminant animals has various metabolic effects [6]. In the body, ALA is converted into docosahexaenoic acid (DHA) and EPA, precursors of 3-series prostaglandins that inhibit cyclooxygenase activity and PGF2α synthesis [2]. Unlike ALA, LA is converted into dihomo-gammα-linolenic acid, a precursor of 1-series prostaglandin and thus arachidonic acid synthesis in the cyclooxygenase pathway [36]. Arachidonic acid is used as a precursor for synthesizing the 2-series prostaglandin (i.e., PGF2-α), which triggers regression of the corpus luteum and thus stimulation of the growth and development of new follicles that eventually increase the secretion of estradiol [37,38]. However, excessive LA supplementation inhibits PGF2-α synthesis through inhibition of the delta-6-desaturase and cyclooxygenase activities [39]. These ­mechanisms can be a possible cause of the low plasma estradiol concentration during estrus in does treated with ALA and LA, even though these animals were still in good condition during estrus. The low plasma estradiol concentrations recorded in this study were still higher than those reported by Gaafar *et al*. [40] in Damascus goats in the estrous cycle (57.1±18.4 pg/mL). Although a low estradiol concentration during estrus may decrease the expression of estrous signs and uterus growth [41], it has the potential to prolong the life span of the corpus luteum [39], as evidenced by the lower estradiol with higher progesterone concentrations in the ALA group. Our results are similar to those of a study conducted by Mahla *et al*. [42], who found that ALA supplementation significantly decreased the estradiol concentration in goats (*Capra hircus*).

The high plasma estradiol concentration in the LAURIC group during estrus was strongly positively associated with a high total number of follicles (Table-5). Sullivan *et al*. [43] reported that each follicle, including the small ones, plays a role in producing estradiol, with the small and medium follicles producing average estradiol concentrations of 0.77 and 2.53 ng/mL, respectively. The high estradiol concentration during estrus in the LAURIC group is related to high estrous behavior [44]. A high level of estradiol secretion during estrus at the beginning of the reproductive process has a strong effect in stimulating uterus growth and development to support the uterine environment for fertilization and conceptus development [45,46].

However, the high concentration of plasma estradiol in the LAURIC group was not followed by improved follicle growth and development for reaching the ovulation stage. Follicle development in the LAURIC and LA groups was not as good as that in the ALA group with its higher number of large preovulatory follicles. LAURIC does not have any anti-inflammatory effects, whereas LA (as a precursor of 2-series prostaglandins) can elicit a pro-inflammatory effect because it can be converted into arachidonate [6]. Therefore, supplementation with lauric and LAs will promote a higher chance of apoptosis due to the lower levels of intracellular glutathione [47] and increased levels of reactive oxygen species (ROS) generated [48]. A high ROS concentration will disturb cellular processes, such as oocyte maturation, and trigger oxidative stress and apoptosis [49]. Oxidative damage of lipids in the oocytes will deteriorate the quality of the cells [50]. By contrast, ALA can reduce the level of ROS [51], thereby reducing the rate of apoptosis and allowing oocyte maturation, growth, and development into the large follicles. ALA can be converted into EPA and DHA, which are the precursors of 3-series prostaglandin and therefore have anti-inflammatory effects [6,52]. This mechanism could explain how ALA increases the number of large follicles. By contrast, despite the high number of medium follicles found in the does treated with LAURIC, the follicles did not develop as well as those in the animals treated with ALA.

The finding of a high number of large preovulatory follicles in the does supplemented with ALA is corroborated by the previous findings in goats receiving omega-3 fatty acid from fish oil [42]. Furthermore, the treatment of cows with omega-3 sourced from flaxseed also increased the number of large follicles [53]. The increased number of large preovulatory follicles in the ALA group could be related to the stimulatory effect of ALA on insulin-like growth factor 1 (IGF-1) synthesis by granulosa cells [54]. IGF-1 is the key regulator of follicular differentiation and other reproductive functions. In particular, it suppresses the apoptotic process in ovarian follicles, thereby reducing the occurrence of atresia and increasing the number of ovulating follicles [55]. In ruminants, fat intake enhances the secretion of insulin, the hormone that facilitates the transfer of glucose as an energy source into ovarian cells [34]. The increased insulin concentration may play a role in mediating the increase of follicle growth either directly through its own receptor or indirectly through its modulation of IGF-1 production by granulosa cells [54]. ALA can increase IGF-1 synthesis and secretion to stimulate the growth and development of follicles [56].

The large preovulatory follicles develop into a large corpus luteum after ovulation. However, the diameters of all classes of follicles in the three groups were similar after fatty acid flushing. Similar results were reported in high-producing dairy cows treated with different types of fatty acids [57]. The higher number of large follicles in does supplemented with ALA should lead to the production of a higher number of progesterone molecules, which will improve the reproductive performance of the animals [58]. Although the diameters of the corpus luteum were similar among all three groups, the measurements in the ALA group tended to be higher at the time of ovulation. As a precursor of 3-series prostaglandin, ALA enhances the growth and development of the corpus luteum, leading to better development of the luteal cells [42]. Dietary supplementation with omega-3 fatty acids reportedly induced the proliferation of granulosa cells, which stimulated enlargement of the corpus luteum [59].

The non-significant differences in the diameters of the corpus luteum observed on days 20 and 21 of flushing were likely because the development of this structure was still in the early stage (i.e., the corpus hemorrhagicum stage) and had not yet reached optimum growth. Therefore, the diameter did not reflect that of a mature corpus luteum, as indicated by the low plasma progesterone concentration (i.e., <1 ng/mL). The corpus luteum produces >1 ng/mL progesterone after 5 days of estrus onset [60]. A low progesterone concentration in the early luteal phase has similarly been reported in small ruminants supplemented with different fatty acids [2,3,42]. The non-significant effect of ALA on the plasma progesterone levels of the three groups in this study may be attributed to their similar corpus luteum diameters, since this transitory gland is responsible for the synthesis and release of the hormone [61].

Despite the finding that the plasma progesterone concentrations were similar (p>0.05) in all three groups, the animals supplemented with ALA tended to have the highest level of this hormone (p=0.34) and the highest corpus luteum diameter (p=0.68). According to the correlation analysis, the plasma progesterone concentration had a statistically significant moderate positive correlation with the corpus luteum diameter, as was also reported by Ishak *et al*. [62] in their study of mares. Does supplemented with ALA had twice the plasma progesterone concentration of does supplemented with LA (Table-4), which is supported by the known role of ALA in inhibiting PGF2-α synthesis and increasing progesterone synthesis [6]. Progesterone is the hormone that stimulates uterine growth and development to support zygote implantation and embryonic growth and development [63]. The plasma progesterone concentration during pregnancy was also consistently significantly higher in ewes treated with ALA -rich flaxseed oil than in those treated with linolenic acid-rich sunflower oil [2].

## Conclusion

The 21-day flushing supplementation of different oils to does with an initial BCS of ≤2 was effective in increasing their BCS to stimulate entry into the reproductive process. The type of fatty acid supplemented in the flushing diet did have various effects on estradiol synthesis and follicle growth and development during estrus. The highest plasma estradiol concentration was found in does supplemented with LAURIC, an SFA from coconut oil; however, it was not followed by sufficient improvement of follicle growth and development to reach the ovulation stage. By contrast, supplementation with ALA, a PUFA from flaxseed oil, stimulated the highest number of large-sized preovulatory follicles.

## Authors’ Contributions

PN, KGW, DAA, and WM designed the concept of research. All authors conducted data interpretation and edited the manuscript. PN contributed to the whole experimental activity, data collection and analysis, and article writing and publication. KGW led this study and contributed to the scientific discussion. DAA provided experimental material and contributed to field observation and scientific discussion. WM assisted with manuscript preparation and discussion. All authors read and approved the final manuscript.
